# Three-dimensional model of normal human dermal tissue using serial tissue sections

**DOI:** 10.3389/fbioe.2024.1347159

**Published:** 2024-03-06

**Authors:** Peng Liu, Tao Zhang, Yihui Huang

**Affiliations:** ^1^ Department of Burn and Plastic, Guangzhou Red Cross Hospital, Medical College, Jinan University, Guangzhou, China; ^2^ Department of Pediatric Medicine, Guangzhou Red Cross Hospital, Medical College, Jinan University, Guangzhou, China

**Keywords:** 3D structure, serial histological sections, dermis, image registration, porosity, average pore diameter

## Abstract

**Background:** This study aims to construct a three-dimensional model of skin dermis utilizing continuous tissue sections, with the primary objective of obtaining anatomical structure data for normal human dermal tissues.

**Methods:** Normal skin tissue specimens were acquired, paraffin-embedded, and subjected to HE staining. Panoramic images of skin sections were captured using a microscope. Tissue section images were aligned using the SIFT and StackReg image alignment methods, with analysis conducted using the OpenCV module. Mimics17 software facilitated the reconstruction of the skin dermal 3D model, enabling the calculation of dermal porosity and the void diameter.

**Results:** Panoramic skin slices exhibited high-resolution differentiation of dermal fibers and cellular structures. Both SIFT and StackReg image alignment methods yielded similar results, although the SIFT method demonstrated greater robustness. Successful reconstruction of the three-dimensional dermal structure was achieved. Quantitative analysis revealed a dermal porosity of 18.96 ± 4.41% and an average pore diameter of 219.29 ± 34.27 μm. Interestingly, the porosity of the dermis exhibited a gradual increase from the papillary layer to the fourth layer, followed by a transient decrease and then a gradual increase. The distribution of the mean pore diameter mirrored the pattern observed in porosity distribution.

**Conclusion:** Utilizing the continuous skin tissue slice reconstruction technique, this study successfully reconstructed a high-precision three-dimensional tissue structure of the skin. The quantitative analysis of dermal tissue porosity and average pore diameter provides a standardized dataset for the development of biomimetic tissue-engineered skin.

## 1 Introduction

Autologous dermal grafts and flaps are commonly employed for wound repair; however, they are associated with clinical challenges such as dermal contracture, scar hyperplasia, and impaired appearance and function (Barbara et al., 2022; Feng and Qiang, 2021; Núria and Joan, 2023). The structural integrity of the dermis plays a crucial role in enhancing skin elasticity, flexibility, and cushioning ability, thereby mitigating contracture and scar formation. Consequently, tissue-engineered skin has emerged as a research hotspot in wound repair (Cuesta et al., 2023; Shila et al., 2023; Youngnam et al., 2024).

Tissue-engineered skin represents a significant approach to effectively address wounds, ameliorate skin contractures, reduce scarring, and enhance clinical outcomes. The characterization of tissue-engineered skin is vital as it influences the physicochemical properties of the skin and impacts the proliferation, differentiation, and tissue regeneration of fibroblasts, as well as the growth of blood vessels and nutrient penetration (Maryam and Bita, 2023; Narayanan et al., 2023; Sarah et al., 2023; Taghizadeh et al., 2023). The design of viable tissue-engineered skin necessitates a foundation in the anatomical structure of the human dermis. Tissue-engineered skin that closely mimics the composition and microstructure of normal skin exhibits superior efficacy in promoting wound healing. However, current tissue-engineered skin lacks essential data pertaining to normal skin, such as dermal fiber composition, porosity, pore diameter, and pore wall thickness (Maria et al., 2022; Ewa et al., 2023a). Therefore, a comprehensive quantitative and qualitative analysis of the micro 3D structure of the skin is beneficial for constructing biomimetic tissue-engineered skin.

The optimal spatial distribution of simulated restored skin tissue cells and extracellular matrix serves as the foundation for the regenerative repair of skin defects. Traditional 3D reconstruction methods such as CT and MRI have low resolution for skin tissue structure, making it difficult to recognize the skin epidermis and dermal fiber structure in the images. However, skin tissue section images provide a clear resolution of dermal fiber and cellular structures, thereby representing the microscopic three-dimensional structure of the tissue (Muro and Akita, 2023). This study focused on reconstructing the three-dimensional anatomical structure of skin tissue based on continuous skin tissue sections.

The key to 3D reconstruction of continuous skin tissue slices lies in the acquisition of high-resolution panoramic images of the sections and accurate alignment of the continuous images. The current literature indicates that multiple small-field-of-view images can be acquired on a continuous basis by these images of tissue sections through an optical microscope and then stitching the images together to form a complete panoramic image of the section. However, this method has disadvantages of low stitching accuracy and a heavy workload. In addition, the existing literature describes manual methods that align continuous tissue section images with low alignment accuracy, making it difficult to quickly and accurately align a large amount of image data (Kiemen et al., 2022; Chang et al., 2023). Our study used a new technical method of panoramic imaging and automatic alignment technology to reconstruct the three-dimensional anatomical structure of skin tissues. This new method improves the accuracy rate and work efficiency, and it lays a foundation for the three-dimensional reconstruction of skin tissues.

## 2 Materials and methods

### 2.1 Materials

The study involved the collection of whole skin from the outer thigh of normal adults, ensuring that the collection site was devoid of any visible injuries or skin diseases. Skin tissue specimens were fixed and preserved in a 10% formalin fixative solution. We placed the fixed specimen in an alcohol gradient for dehydration, followed by xylene for clarification. The skin specimen was trimmed into a 1 cm × 1 cm skin tissue block, with depth to subcutaneous, and was then paraffin-embedded and HE-stained. The study protocol was approved by the Institutional Review Board of Guangzhou Red Cross Hospital (APPROVAL NUMBER/2023-009-01) on 15 February 2023.

### 2.2 Acquisition of continuous skin tissue section images

Each section was scanned using a Motic BA600 Mot-7.5 fully automatic microscope. Panoramic tissue sections were generated, and the resulting images were stored in JPG format. The region of interest was carefully selected for subsequent analysis.

### 2.3 Automatic alignment of continuous skin tissue section images

Continuous tissue section images often exhibit deviations such as translation and rotation. To address this, the images of skin tissue sections underwent alignment processing. SIFT and StackReg are image registration plugins used in ImageJ software. These can quickly align a series of image slices. Each image slice is used as a template for aligning the next slice. These two image alignment methods have been successfully used in industrial applications and in the aerospace and medical fields. In this study, continuous tissue section images were imported into ImageJ software, and the SIFT and StackReg plugins were used to quickly and automatically align continuous section images by selecting the “Similarity” mode. Ten randomly selected groups of section images were used to assess the effectiveness of image alignment. The OpenCV tool was used to calculate the root-mean-square error (MSE), structural similarity score (SSIM), and mutual information measure (MI) value of the two image alignment methods. Statistical analysis was then conducted to compare and evaluate the alignment effects of the two methods.

### 2.4 Image segmentation of skin tissue sections

Following HE staining, the skin tissue structure became clearly distinguishable, with the gray value of dermal fibers exhibiting a noticeable contrast against other tissue structures in the gray image. Leveraging the threshold segmentation method, the epidermal and dermal fiber structures of the skin tissue were accurately segmented. Upon importing continuous skin tissue section images into Mimics20.0 software and ensuring precise alignment, the threshold segmentation tool was employed. Grayscale values of the skin tissue section were adjusted until the desired skin tissue structure was selected. Any missed portions were manually edited for segmentation refinement.

### 2.5 3D reconstruction of skin tissue structure

**FIGURE 1:**
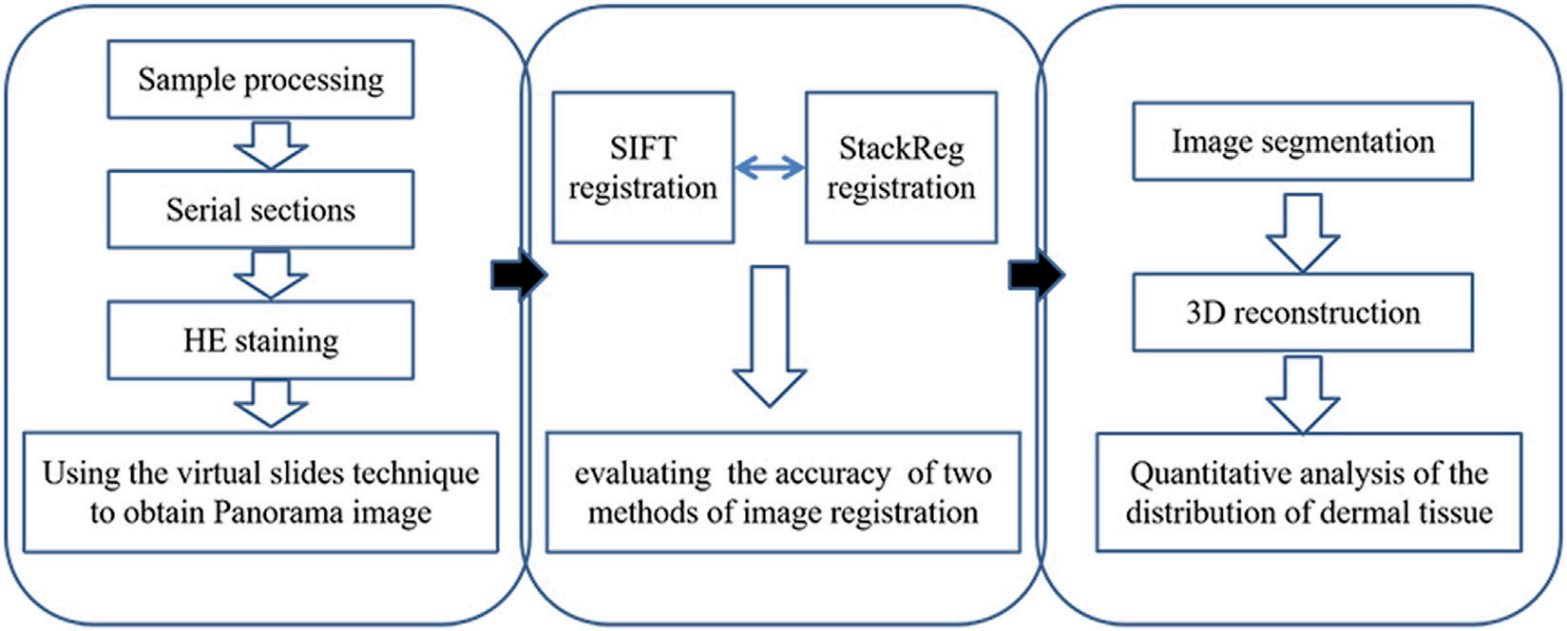
Flowchart of experimental design.

Next, skin tissue slices were imported into Mimics20.0 software, and the epidermal and dermal structures were segmented, generating corresponding binary images. The 3D reconstruction tool was then utilized to select the optimal quality 3D reconstruction through contour interpolation. Sequentially, 3D reconstruction based on surface drawing was performed for each skin tissue structure, resulting in the visualization of the 3D model representing the skin tissue structure. A flowchart of the experimental design shows the process of panorama imaging, image registration, and three-dimensional reconstruction (Figure 1).

### 2.6 Quantitative analysis of the distribution of dermal tissue porosity and average pore diameter size

A total of 100 consecutive skin tissue sections were randomly chosen, and the dermal structure was divided equally into 10 layers. These sections were imported into Mimics20.0 software. Within this software, 60 regions of interest (ROIs) were randomly selected for further analysis. The Tissue Engineering Analysis Module in Mimics20.0 was then employed to perform quantitative analyses on the selected regions of interest: dermal tissue porosity and average pore diameter size were measured, providing valuable insights into the microstructural characteristics of the dermal tissue.

### 2.7 Statistical analysis

Pair t-testing was used to assess the accuracy of the two methods of image registration. All the data were analyzed using SPSS statistical software, version 27.0 (SPSS, Inc.). The level of significance was *p* < 0.05 (two-sided).

## 3 Results

### 3.1 HE staining results

**FIGURE 2:**
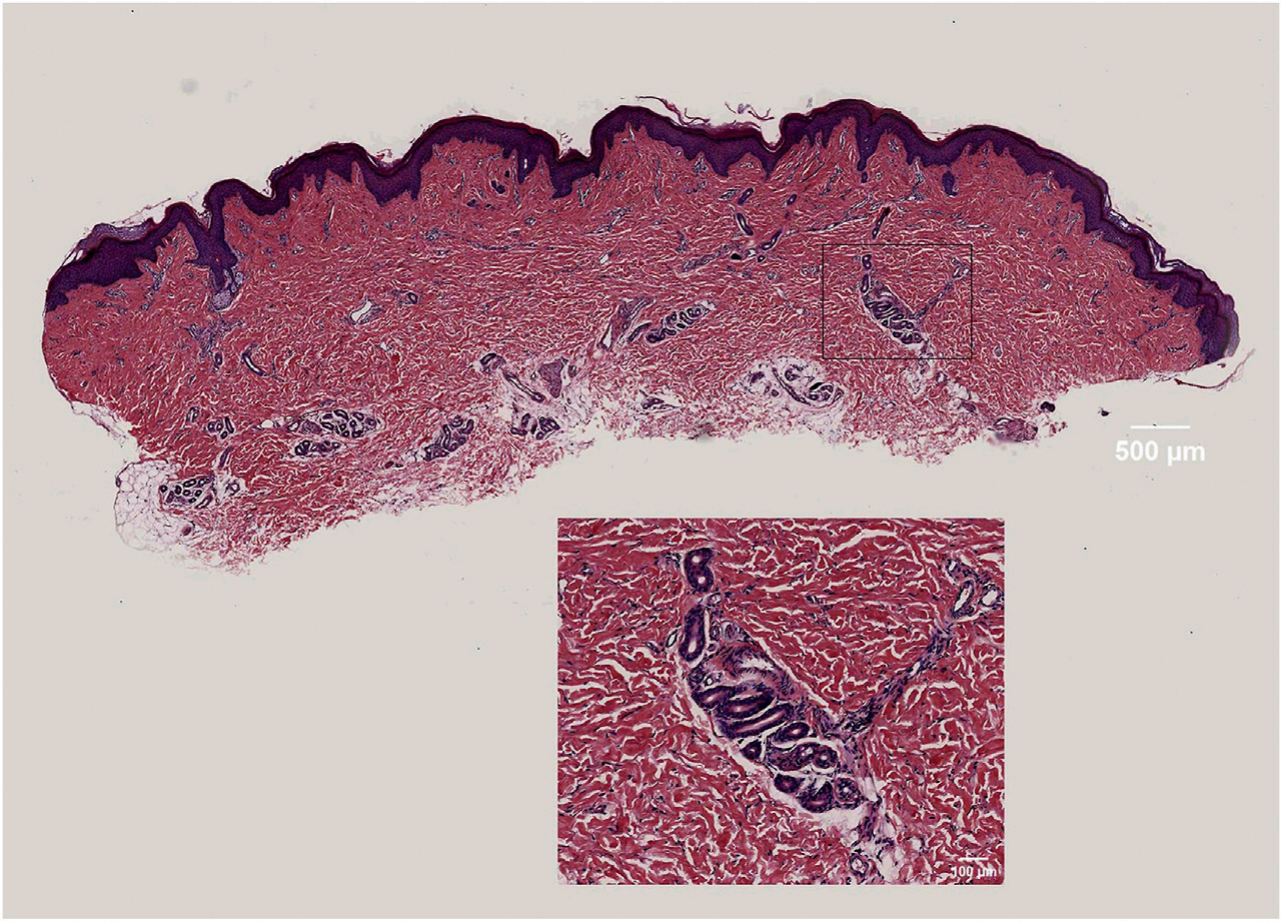
High-resolution display of dermal fibers, fibroblasts, and sweat gland structures in skin tissue.

The panoramic image of the tissue section, following HE staining, exhibits high resolution and provides a detailed view of the epidermis, dermis, hair follicles, and sweat glands. This image accurately outlines the cellular structure of the epidermis and dermis, as well as the dermal fiber structure. These findings serve as the foundation for the subsequent three-dimensional reconstruction of the skin tissue structure (Figure 2).

### 3.2 Quantitative comparison of alignment effects

**FIGURE 3:**
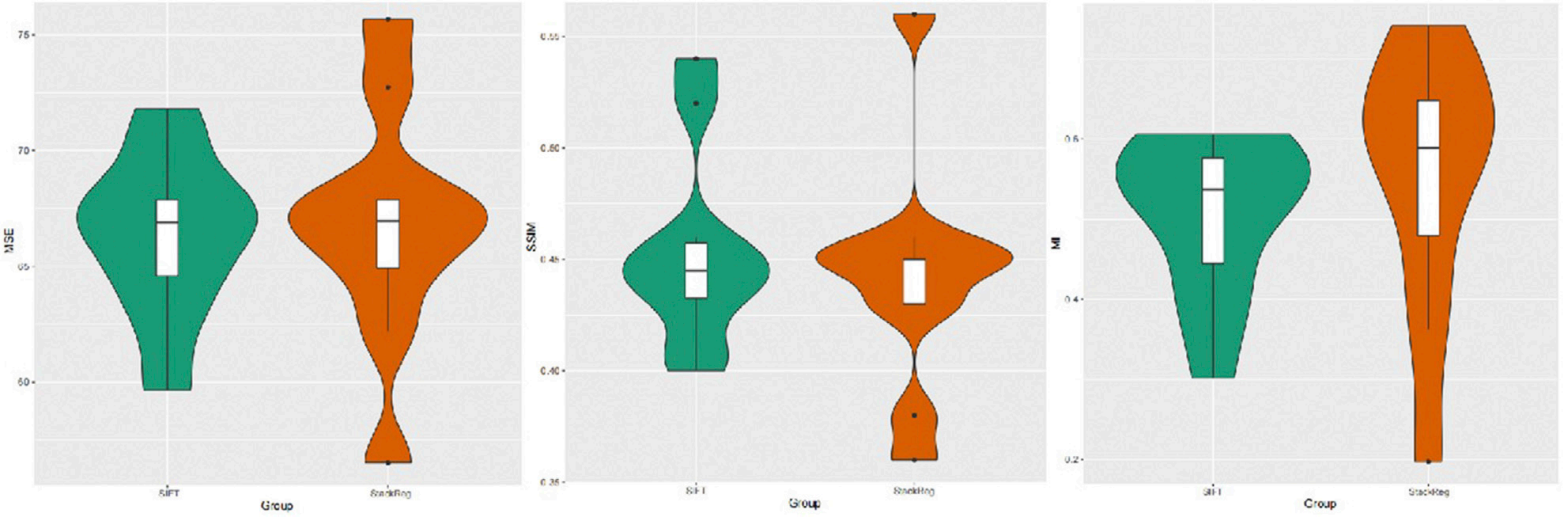
Image mean square error, mutual information measure, and structural similarity scores obtained by the SIFT and StackReg alignment methods.

A quantitative analysis of the alignment effects was conducted by statistically evaluating the mean square error, mutual information, and structural similarity scores of two image alignment methods. The results indicate that the mean square error for images aligned using the SIFT method was 66.319, while, for images aligned with StackReg, it was 66.727. The mean mutual information value for SIFT-aligned images was 0.5062054, and for StackReg-aligned images, it was 0.5495679. Additionally, the mean structural similarity score for SIFT-aligned images was 0.454, while, for StackReg-aligned images, it was 0.442. A paired t-test was performed, yielding a *p*-value greater than 0.05. This suggests that the performance of the two alignment methods is comparable. However, it is noted that the SIFT algorithm demonstrates greater stability than the StackReg-based alignment method (Figure 3).

### 3.3 Three-dimensional structure of skin tissue

**FIGURE 4:**
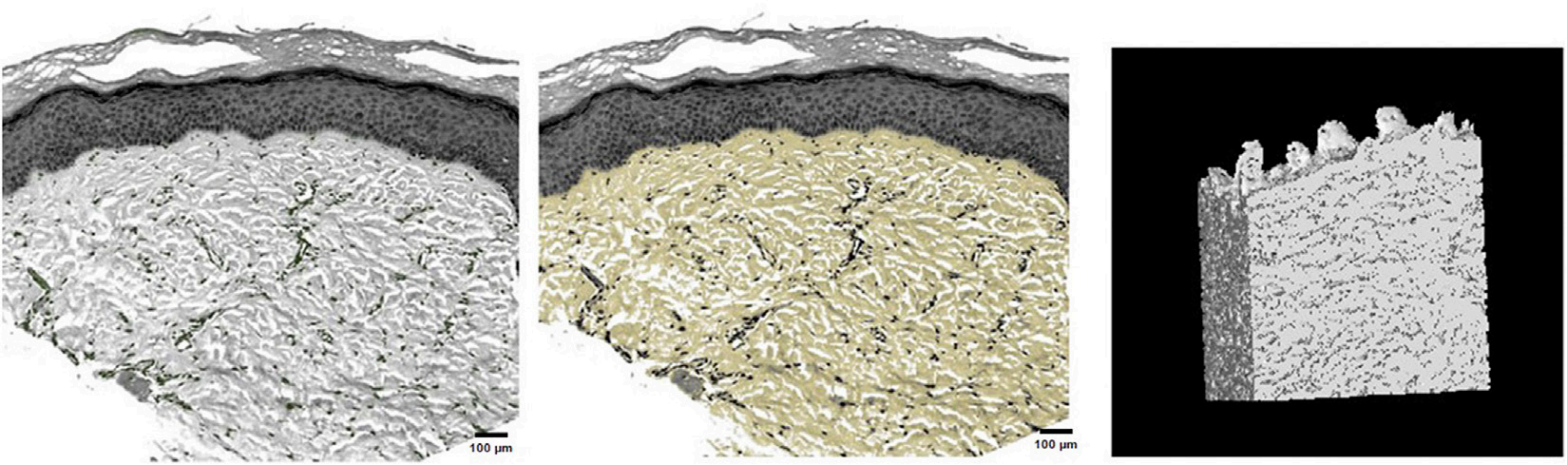
Fibroblast distribution in dermal tissue, precise selection of dermal fibers, and three-dimensional reconstruction of dermal tissue.

This study successfully identified dermal fibers and fibroblasts, allowing for the clear three-dimensional reconstruction of the dermal tissue structure. This reconstruction provides a vivid representation of the three-dimensional spatial image, illustrating that dermal fibers within the skin’s dermis were scattered. These fibers interweave to form pores of varying sizes, with each pore containing differing numbers of fibroblasts (Figure 4).

### 3.4 Distribution pattern of porosity and average pore diameter in dermal tissue

**FIGURE 5:**
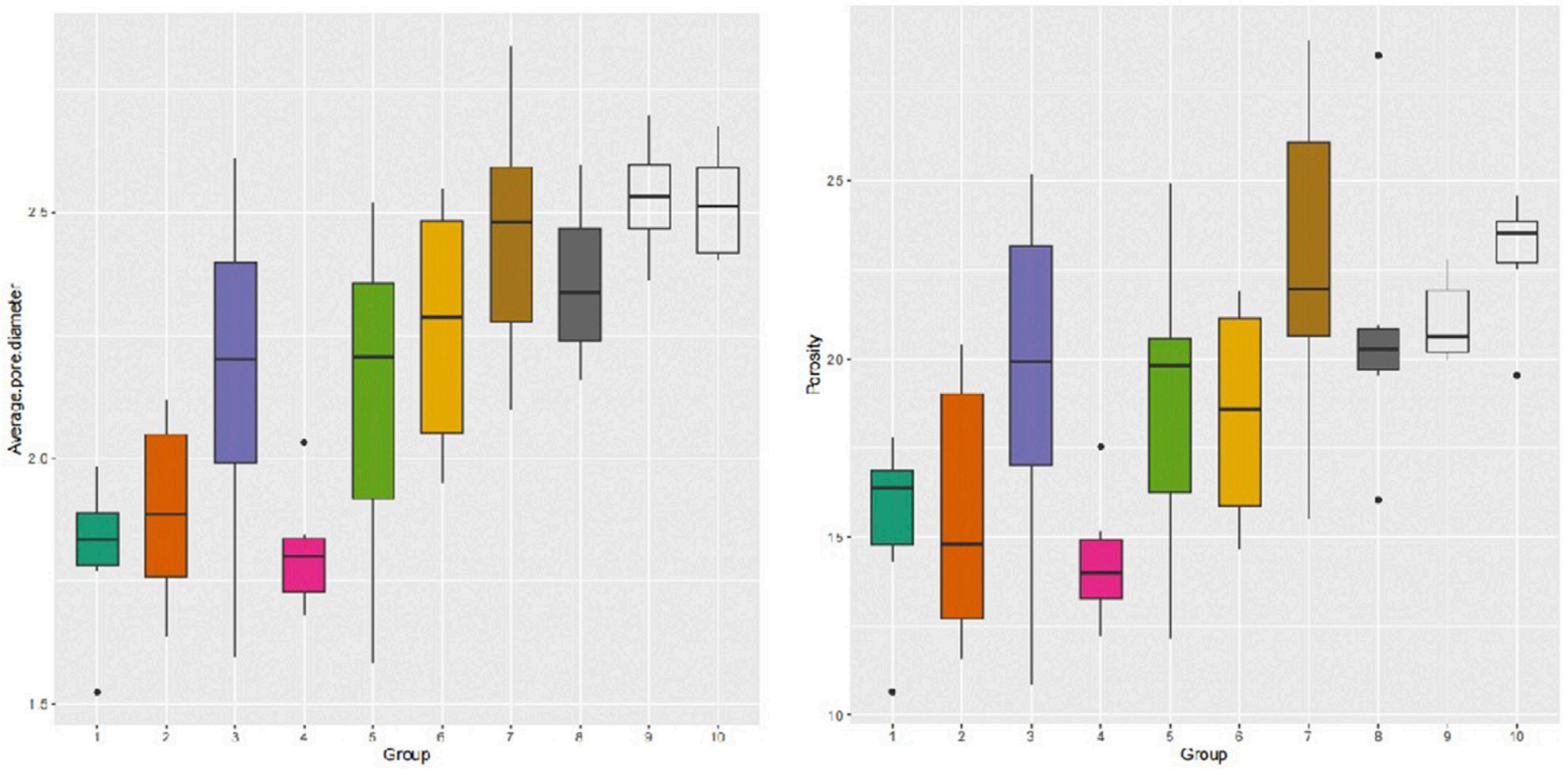
Distribution of longitudinal dermal mean pore diameter and porosity.

A longitudinal quantitative analysis of dermal porosity and average pore diameter in human skin tissues was conducted. The results revealed that dermal porosity gradually increased from the papillary layer, decreased in the fourth layer, and then exhibited a gradual increase once again. Furthermore, the distribution of the mean pore diameter mirrored the pattern observed in the distribution of porosity (Figure 5). The site of interest was randomly selected and subjected to statistical analysis, indicating a dermal porosity of 18.96% ± 4.41% and an average pore diameter of 219.29 ± 34.27 μm in human skin tissues.

## 4 Discussion

Tissue-engineered skin has emerged as an optimal method for repairing tissue defects, with current research emphases on scaffold materials, seed cells, and tissue construction (Sorour et al., 2021; Jie et al., 2022; Robab et al., 2022). The construction of tissue-engineered skin for mending skin tissue defects has evolved into a research hotspot, offering innovative possibilities for tissue repair. Tissue-engineered dermal scaffolds play a pivotal role by providing a three-dimensional environment conducive to cell adhesion, growth, and differentiation. Notably, the biomimicry of tissue-engineered dermal scaffolds significantly influences the outcomes of wound repair. Crucial parameters in scaffold materials include skin dermal tissue porosity distribution and average pore diameter. The acquisition of these parameters from normal human skin dermal tissues is instrumental in the development of artificial dermal scaffold materials (Nupur et al., 2021; Yawen et al., 2021; Ewa et al., 2023b). The three-dimensional reconstruction of skin dermis, based on continuous tissue slices, proves to be a valuable approach for obtaining and understanding these essential parameters.

Porosity stands out as a crucial parameter in tissue engineering materials, exerting a notable impact on fluid transport properties within the dermis (Bohumila et al., 2023; Jeyachandran and Cerruti, 2023; Rasha et al., 2023). The three-dimensional structure of decellularized allogeneic dermis, reconstructed using micro-CT, yielded a calculated porosity of 68.3 ± 5.8% (Wang et al., 2015). However, our reconstruction of the 3D structure of normal human dermis through continuous tissue sectioning techniques revealed a dermal porosity of 18.96 ± 4.41%. This discrepancy may be attributed to significant skin shrinkage post-ex vivo, resulting in reduced porosity.

Another pivotal parameter in tissue engineering materials is the mean pore diameter, which influences cell adhesion and migration. It is now understood that fibroblasts in the dermis deposit within pores composed of dermal fibers. Smaller pore diameters in dermal scaffolds pose challenges for fibroblast adhesion and migration in dermal pores (Ghazal et al., 2022; Zohaib et al., 2022; Olevsky et al., 2023). Our reconstruction of the three-dimensional structure of normal human dermis through continuous tissue sectioning techniques yielded an average pore diameter of 219.29 ± 34.27 μm. Therefore, it is imperative that the pore diameter for constructing dermal scaffolds exceeds the diameter of fibroblasts. The reconstruction of the three-dimensional dermal structure using continuous tissue sectioning techniques also revealed that the distribution of dermal scaffold pores lacks an obvious pattern.

Image alignment is pivotal in reconstructing the three-dimensional structural features of human skin dermis, where the quality of alignment directly influences the accuracy of the reconstructed features. Continuous skin tissue slices exhibit variations in grayscale attributes, position (translation and rotation), scale, and nonlinear deformation. Previous studies on image alignment have only paid attention to translation and rotation while ignoring the existence of transformations such as scale expansion and deformation in tissue section images (Liu et al., 2018). In this study, we optimized the global image alignment on the basis of previous studies and used the Similarity alignment mode to reduce the misalignment caused by image deformation. These methods improved the accuracy of the data and produced data that were more representative of the real dermal porosity and pore diameter. The SIFT algorithm, recognized as an excellent feature alignment operator, can be harnessed to address these challenges (Li et al., 2015; Erbing et al., 2023; Meng et al., 2023). Key parameters for evaluating image alignment efficacy include mean square error, mutual information, and structural similarity score. In this study, a statistical analysis of these parameters for two image alignment methods revealed that the SIFT algorithm-based method is comparable in effectiveness to the StackReg image alignment method but demonstrates higher stability.

Current natural and degradable chemically synthesized polymeric materials used in constructing tissue-engineered skin scaffolds fall short of truly mimicking the structural and mechanical properties of the dermis. However, the use of decellularized dermal graft materials has shown excellent clinical results in repairing skin tissue defects. Therefore, understanding the structural features of human skin dermis is crucial for guiding the construction of biomimetic tissue-engineered skin.

The reconstruction of the three-dimensional microstructure of skin dermal tissue, based on serial skin tissue sections, coupled with quantitative analysis at the tissue or cellular level, provides a standard dataset. This dataset is invaluable for advancing the construction of biomimetic tissue-engineered skin and enhances our ability to replicate the intricate features of human skin dermis in engineered substitutes.

## 5 Conclusion

By utilizing the continuous skin tissue slice reconstruction technique, this study successfully achieved a high-precision three-dimensional reconstruction of skin tissue structure. The quantitative analysis of dermal tissue porosity and average pore diameter not only enhances our understanding of the microstructural characteristics but also contributes to the establishment of a standard dataset.

## Data Availability

The original contributions presented in the study are included in the article/Supplementary material; further inquiries can be directed to the corresponding author.
